# Complete Genome Sequence of *Anoxybacillus flavithermus* Strain 52-1A Isolated from a Heat-Processed Powdered Milk Concentrate

**DOI:** 10.1128/genomeA.00800-17

**Published:** 2017-08-10

**Authors:** Taurai Tasara, Marina Morach, Jochen Klumpp, Roger Stephan

**Affiliations:** a Institute for Food Safety and Hygiene, Vetsuisse Faculty University of Zurich, Zurich, Switzerland; b Institute of Food, Nutrition and Health, ETH Zurich, Zurich, Switzerland

## Abstract

The thermophilic spore-forming bacterium *Anoxybacillus flavithermus* is responsible for powdered milk product spoilage, and its presence in dairy processing environments is a concern. Here, the complete genome sequence of the *A. flavithermus* strain 52-1A isolated from a heat-processed powdered milk product concentrate in Switzerland is presented.

## GENOME ANNOUNCEMENT

The contamination of milk powder by spore-forming heat-resistant milk spoilage bacteria remains an important concern for the dairy industry (1). *Anoxybacillus flavithermus* is a thermophilic sporeformer that frequently occurs as a contaminant in dairy processing environments, leading to fouling of powdered milk products (1–3). Only a few *A. flavithermus* strains have had their complete genomes sequenced to date (4–6). An analysis based on this limited number of *A. flavithermus* genomes has shown that this bacterium is harboring genes that are compatible with its lifestyle adaptations, such as high growth temperatures and extreme pH conditions (7). More complete polished *A. flavithermus* genomes are clearly needed in order to enhance the understanding of the genetic and metabolic diversity as well as physiological mechanisms involved in adaptation of this bacterium to dairy production environments. We have determined the complete genome sequence of the *A. flavithermus* 52-1A strain, which was isolated from a heat-processed powdered milk product concentrate with an off flavor in Switzerland.

Genomic DNA was isolated from *A. flavithermus* 52-1A with the GenElute bacterial genomic DNA kit (Sigma, Buchs, Switzerland) and sequenced using the Pacific Biosciences single-molecule real-time (SMRT) sequencing technology at the Functional Genomics Centre of the University of Zurich. Sequencing generated 47,773 sequence reads (129-fold genome coverage) with an average length of 7,219 kb. The sequence reads were assembled *de novo* using SMRT Analysis 2.3.0 software and the HGAP3 algorithm. This sequence assembly generated a complete *A. flavithermus* genome sequence of 2.8 Mb comprising one chromosome (2,805,288 bp) and one plasmid (24,836 bp). Annotation of the genome was carried out using the NCBI Prokaryotic Genome Annotation Pipeline (PGAP) (http://www.ncbi.nlm.nih.gov/genome/annotation_prok/) (8). Overall, there were 3,013 genes identified in the *A. flavithermus* 52-1A genome. Of these, 2,985 genes are on the chromosome, whereas 28 genes are plasmid encoded. The *A. flavithermus* 52-1A chromosome has a 42.2% GC content and carries 2,869 coding sequences, 219 pseudogenes, 85 tRNA genes, and 9 rRNA operons. Using the phage search tool PHAST, the *A. flavithermus* 52-1A genome was predicted to have a single prophage region located at positions 1556241 to 1583498 (27,275 bp) (9). Genome comparison with other available *A. flavithermus* genomes shows that the 52-1A strain is most closely related to the *A. flavithermus* TNO-09.006 strain, sharing 99.28% average nucleotide identity.

### Accession number(s).

Genome sequences for the *A. flavithermus* 52-1A chromosome and plasmid are available in GenBank under accession numbers CP021838 and CP021839, respectively.
